# Case report: Novel NIPBL-BEND2 fusion gene identified in osteoblastoma-like phosphaturic mesenchymal tumor of the fibula

**DOI:** 10.3389/fonc.2022.956472

**Published:** 2023-01-05

**Authors:** Tomohisa Sakai, Yusuke Okuno, Norihiro Murakami, Yoshie Shimoyama, Shiro Imagama, Yoshihiro Nishida

**Affiliations:** ^1^ Department of Orthopedic Surgery, Nagoya University Graduate School of Medicine, Nagoya, Japan; ^2^ Rare Cancer Center, Nagoya University Hospital, Nagoya, Japan; ^3^ Department of Virology, Nagoya City University Graduate School of Medical Sciences, Nagoya, Japan; ^4^ Department of Pediatrics, Nagoya University Graduate School of Medicine, Nagoya, Japan; ^5^ Department of Pathology and Laboratory Medicine, Nagoya University Hospital, Nagoya, Japan; ^6^ Department of Rehabilitation, Nagoya University Hospital, Nagoya, Japan

**Keywords:** phosphaturic mesenchymal tumor, tumor induced osteomalacia, bone tumor, RNA sequencing, fusion gene, whole exome sequencing

## Abstract

Phosphaturic mesenchymal tumor (PMT) is a rare tumor that secretes fibroblast growth factor 23 (FGF23) and causes hypophosphatemia and tumor-induced osteomalacia (TIO). Fusion genes *FN1-FGFR1* and *FN1-FGF1* have been detected in some PMTs, but the pathogenesis of PMTs without these fusion genes remains unclear. Here, we report a 12-year-old boy with persistent muscle weakness and gait disturbance. Roentgenographic examination revealed a radiolucent lesion with endosteal scalloping in the left fibula, while his serum level of FGF23 was markedly increased. Combined with simple X-ray findings of other body parts, we suspected that TIO was caused by PMT, and resected the tumor. After resection, the serum level of FGF23 started to decrease immediately and normalized within 3 hours after resection, with this being earlier than normalization of the serum phosphorus level. In RNA sequencing, *FN1-FGFR1* and *FN1-FGF1* were not detected, but a novel *NIPBL-BEND2* fusion gene was identified. When we forcedly expressed this fusion gene in HEK293T cells and MG63 cells, cell proliferation was enhanced in both cell lines. Furthermore, Gene set enrichment analysis of HEK293T cells showed significant upregulation of MYC-target genes. Our results suggest that this novel *NIPBL-BEND2* fusion gene promotes cell proliferation possibly *via* the MYC pathway and might be one of the etiologies of PMTs other than *FN1-FGFR1* or *FN1-FGF1*.

## Introduction

Phosphaturic mesenchymal tumor (PMT) is an extremely rare neoplasm that causes tumor-induced osteomalacia (TIO) in most affected patients, usually through the production of fibroblast growth factor 23 (FGF23) (1, 2). In 1987, the term “phosphaturic mesenchymal tumor” was first proposed and classified into four morphological variants: mixed connective tissue, osteoblastoma-like, nonossifying fibroma-like, and ossifying fibroma-like (3). It was also reported that PMT is the most common cause of TIO, accounting for 80% of the total (4). PMT was adopted as a single entity of uncertain differentiation soft tissue tumor in the 5^th^ edition of the WHO 2020 classification (1) However, a significant number of cases also occur in bone.

FGF23 causes hypophosphatemia by inhibiting phosphate reabsorption in proximal tubules of the kidney and phosphate absorption in intestine (5, 6). *FGF23* was initially reported as a gene responsible for autosomal-dominant hypophosphatemic rickets (7), and was found to be also elevated in the serum of patients with TIO (8, 9), thus making measurement of serum FGF23 helpful in the differential diagnosis of hypophosphatemic diseases (10). Patients with TIO typically complain of pain and muscle weakness, but sometimes the diagnosis is delayed due to the tumor’s variable location and often tiny size (11).

The fusion gene in PMT was first reported in 2015, and *FN1-FGFR1* was found in 9 of 15 PMTs (12). Next, the fusion gene *FN1-FGF1* was reported in 2016, with a report showing *FN1-FGFR1* in 42% (21/50) and *FN1-FGF1* in 6% (3/50) of 50 PMTs (13). These fusion genes are thought to promote hypophosphatemia by enhancing the secretion of FGF23 *via* a mutant ligand or a mutant receptor in the FGF1-FGFR1 pathway (12, 13). However, these fusion genes have been identified in fewer than half of PMTs, and the pathogenesis of the other PMTs remains unknown.

Here, we report a case of PMT of the left fibula in a 12-year-old boy. The serial serum level of FGF23 was measured dynamically after surgical resection, and RNA sequencing was also performed to detect any gene mutations.

## Case presentation

**Figure 1:**
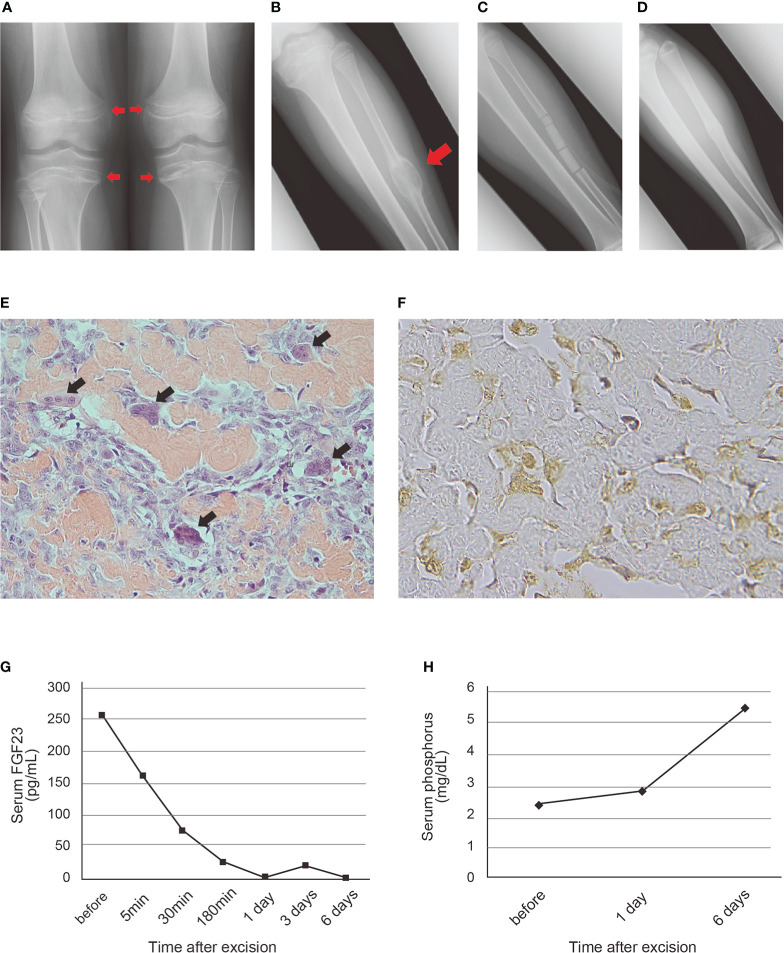
Roentgenographic, histopathological and blood examination findings of the patient with PMT **(A)** Deficiency of mineralization in the epiphysis of the bilateral proximal tibias and distal femurs on admission. **(B)** Radiolucent lesion with endosteal scalloping and marginal sclerosing in the left fibular shaft. A red arrowhead indicates the tumor-like lesion. **(C)** The cavity of the tumor filled with beta-tricalcium phosphate (β-TCP) after resection. **(D)** Good bone formation observed at 21 months after surgery. **(E)** Hematoxylin and eosin staining of the resected specimen. Irregularly deposited osteoid and osteoblast-like tumor cells were scattered between the osteoid. Black arrowheads indicate reactive osteoclastic giant cells (original magnification, x400). **(F)** Immunohistochemical staining of FGF23. Positive staining was observed in the cytoplasm of the osteoblast-like tumor cells (original magnification, x800). **(G, H)** Transition of serum FGF23 **(G)** and phosphorus **(H)** level after excision of the tumor. Normal ranges of serum FGF23 and phosphorus level are <50 pg/mL and 3-4.7 mg/dL, respectively.

A 12-year-old boy with a one-year history of muscle weakness and gait disturbance that progressed slowly presented to our hospital. He had no remarkable past medical history or family history of metabolic bone disease. Roentgenographic examination revealed a deficiency of mineralization like that seen in rickets patients in the epiphysis of the bilateral proximal tibias and distal femurs (**Figure 1A**), and a radiolucent lesion with endosteal scalloping and marginal sclerosing in the left fibula (**Figure 1B**). Blood examination revealed a low serum phosphorus level of 2.3 mg/dL (reference range: 3.0-4.7 mg/dL), and a markedly high serum FGF23 level of 329 pg/mL (reference range: <50 pg/mL), and so we suspected PMT with TIO caused by the tumor-like lesion in the left fibula.

We resected the tumor *en bloc* by preserving the periosteum of the fibula after confirming its benignity by intraoperative frozen section diagnosis, and the cavity of the tumor was filled with beta-tricalcium phosphate (β-TCP) (**Figure 1C** and **Supplemental Figure 1**). The muscle weakness gradually improved, and the gait disturbance normalized within two months. In the postoperative 21-month follow-up, he had no symptoms, and hypophosphatemia was not detected. Roentgenographic examination revealed absorption of the β-TCP and bone formation and union of the fibular shaft (**Figure 1D**).

Histopathological examination of the resected tumor revealed irregularly deposited osteoid and osteoblast-like tumor cells scattered between the osteoid and reactive osteoclastic giant cells (**Figure 1E** and **Supplemental Figure 2**). In immunohistochemistry, expression of FGF23 was shown in the cytoplasm of the osteoblast-like tumor cells (**Figure 1F**), CD56 was diffusely positive on the cell membrane of the tumor cells and SATB2 was diffusely positive on the tumor cells (**Supplemental Figure 2**).

After resecting the tumor, the serum FGF23 level started to decrease immediately and normalized within 3 hours (**Figure 1G**). It was also within the normal range five days after surgery. The increase in serum phosphorus level was slightly delayed as compared with that of the FGF23 level, and was observed 6 days after the operation (**Figure 1H**).

**Figure 2:**
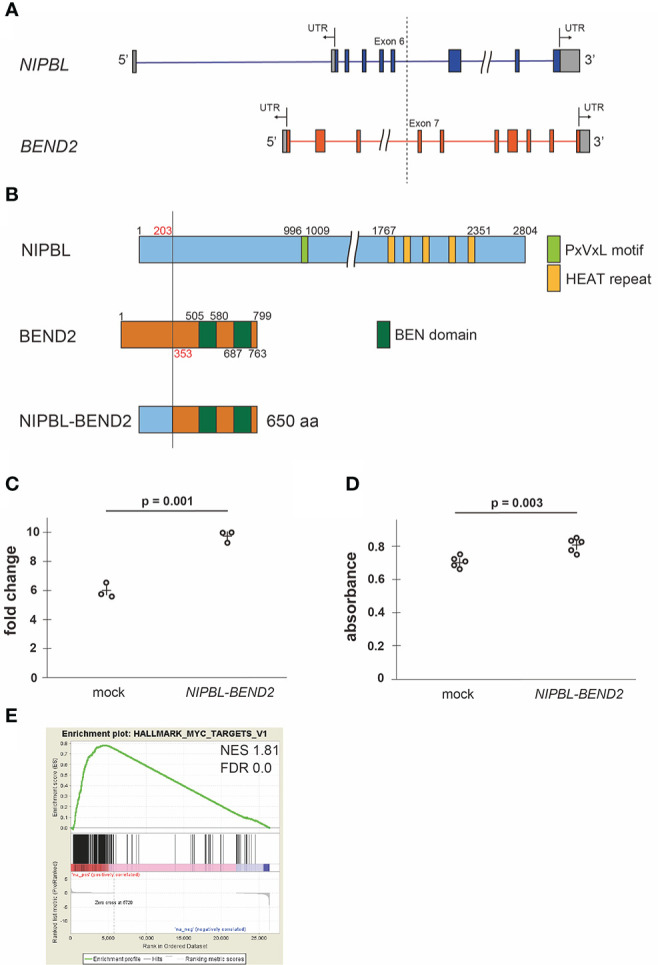
Fusion protein NIPBL-BEND2 and functional analyses **(A)** Structure of the *NIPBL* and *BEND2*. Boxes indicate exons, the gray parts of boxes indicate untranslated region (UTR) and the dotted line indicates genomic breakpoints. The fusion transcript contained exons 1-6 of *NIPBL* and exons 7-14 of *FGFR1*. **(B)** Structure of predicted NIPBL-BEND2 fusion protein. Numbers indicate amino acid residues. **(C)** Cell counts of mock- or *NIPBL-BEND2*- transfected HEK293T cells at 48hr. n=3 each. The plus signs indicate mean values. **(D)** MTS assay of mock- or NIPBL-BEND2- transfected MG63 cells at 48hr. n=5 each. The plus signs indicate mean values. **(E)** Gene set enrichment analysis comparing the expression profiles obtained from mock- and *NIPBL-BEND2-* transfected HEK293T cells. The genes in the HALLMARK_MYC_TARGET_V1 gene set were upregulated in *NIPBL-BEND2*-transfected cells. NES, normalized enrichment score; FDR, false discovery rate.

We performed RNA sequencing using a resected specimen from the patient and identified a novel in-frame fusion involving *NIPBL* (encoding Nipped-B gene product and fungal Scc2-type sister chromatid cohesion proteins) and *BEND2* (encoding a protein which has two BEN domains in the C-terminus) (**Figure 2A**). The fusion protein contained the phosphorylation site derived from *NIPBL* and the BEN domain derived from *BEND2* (**Figure 2B**). Whole-exome sequencing identified three point mutations (IFT172:NM_015662:exon3:c.A263G:p.N88S; VAF = 0.22, GAB2:NM_080491:exon6:c.A1409G:p.D470G; VAF = 0.15, and PRKCH : NM_006255:exon14:c.A1996T:p.I666F; VAF = 0.17), none of which

were reported as driver mutations. In polymerase chain reaction, the amplification of target regions containing breakpoint of the chromosomal structure was confirmed using two primer sets in tumor DNA of the PMT (**Supplemental Figure 3**).

We cloned and transfected the *NIPBL-BEND2* fusion gene to HEK293T and MG63 osteoblast lineage cell line. The *NIPBL-BEND2* transfected cells showed faster proliferation at 48 hours after transfection (p = 0.001 and 0.003, Student’s t-test, respectively). (**Figures 2C, D**). A gene set enrichment analysis of the fusion gene-introduced HEK293T cells identified a significant enrichment of MYC-target genes, consistent with faster proliferation (**Figure 2E**). However, the expression of FGF23 (log2 fold change; 0.031) and FGFR1 (log 2 fold change; 1.59) was not changed significantly by the transfection in addition to the KL/KLB, SPP1, SFRP4, and MEPE (**Supplemental Data 1**), even though the tumor mRNA showed relatively high FPKM in these genes (**Supplemental Table 3**).

## Discussion

The fusion genes *FN1-FGFR1* and *FN1-FGF1* have been reported as causative fusion genes in PMT; however, they account for fewer than half of PMT cases, leaving the other causes of PMT still unclear. In this report, we presented the novel *NIPBL-BEND2* fusion gene in a case of PMT without the two causative fusion genes. This is the first report of a fusion gene other than *FN1-FGFR1* and *FN1-FGF1* in PMT. In *in vitro* experiments, the expression of FGF1-FGFR1 pathway-related genes was not increased in *NIPBL-BEND2*-transfected HEK293T cells. However, the expression of MYC target genes, which has been implicated in cell proliferation, was significantly upregulated by the fusion gene. We also observed faster proliferation in an osteoblast-like cell line after the introduction of *NIPBL-BEND2*. Scattered osteoblast-like tumor cells were observed in the specimens of the present case. Because osteoblasts have been reported to secrete FGF23 (14), the fusion gene expressed in the osteoblast-like tumor cells may be involved in the elevation of serum FGF23 and the development of osteomalacia.

*NIPBL* encodes the homolog of the Drosophila melanogaster Nipped-B gene product and fungal Scc2-type sister chromatid cohesion proteins. The Drosophila protein facilitates enhancer-promoter communication of remote enhancers and plays a role in developmental regulation. It is also homologous to a family of chromosomal adherins with broad roles in sister chromatid cohesion, chromosome condensation, and DNA repair. The best-known result of mutation in *NIPBL* is Cornelia de Lange syndrome (15). NIPBL gene mutations are also associated with malignant neoplasms. The somatic mutations in *NIPBL* have been found in gastric and colorectal cancers and have been reported to be associated with tumorigenesis by altering microsatellite instability (16). The fusion genes involving *NIPBL* have been reported in acute megakaryoblastic leukemia (*NIPBL-HOXB9*) (17) and atypical tenosynovial giant cell tumor (*NIPBL-ERG*) (18), and it is considered that the NIPBL promoter may contribute to changes in the expression of fusion partners (18).

*BEND2* encodes a protein that has two BEN domains in the C-terminus. These domains are found in proteins which participate in protein and DNA interactions occurring during chromatin restructuring or transcription. The fusion genes containing *BEND2* have been reported in neuroepithelial tumors (*MN1-BEND2)* (19) and spinal cord astroblastoma (*EWSR1-BEND2*) (20). However, how BEND2 is related to the tumorigenicities remains unclear. In *NIPBL-BEND2*, the *NIPBL* promoter may alter NIPBL-BEND2 expression and be implicated in cell proliferation as a result. But further research will be needed to verify this.

There are several limitations to this study. First, it is unclear whether *NIPBL-BEND2* is a definitive causal fusion gene of the osteoblastoma-like variant PMT because it is a case report. Secondly, experiments using actual PMT cells have not been performed. Thirdly, mRNA expression analysis has been performed on NIPBL-BEND2 transfected HEK293T cells, but mRNA expression analysis in the actual tumor tissues compared with the normal tissues has not been performed. In addition, although the expression of FGF23 in the tumor specimen has been confirmed by immunohistochemistry, it has not been confirmed by another method including chromogenic *in situ* hybridization (CISH) (21). It is necessary to accumulate more cases of PMTs and performed further research.

## Data availability statement

The original contributions presented in the study are included in the article/**Supplementary Material**. Further inquiries can be directed to the corresponding author.

## Ethics statement

The studies involving human participants were reviewed and approved by 2014-0181. Written informed consent to participate in this study was provided by the participants’ legal guardian/next of kin. Written informed consent was obtained from the minor(s)’ legal guardian/next of kin for the publication of any potentially identifiable images or data included in this article.

## Author contributions

NM, YO and TS performed the research, analyzed the data, and wrote the paper. YN collected clinical samples and information and wrote the paper. YS performed histopathological analyses and wrote the paper. YO, YN and SI cooperatively designed and performed the research, led the project, and wrote the paper. All authors contributed to the article and approved the submitted version.
