# Supplementary material for: Case report: Novel NIPBL-BEND2 fusion gene identified in osteoblastoma-like phosphaturic mesenchymal tumor of the fibula
Source: Front Oncol. 2023 Jan 5;12:956472. doi: 10.3389/fonc.2022.956472 (PMC9850095; doi:10.3389/fonc.2022.956472)
Supplement: Supplementary file 4 [file DataSheet_4.docx]

Supplemental materials

Supplemental Methods pp. 2-4

Supplemental Figures pp. 5-8

Supplemental Table pp. 9, 10

Supplemental Methods

*Immunohistochemistry*

Immunohistochemical staining was performed using anti-FGF23 antibody (polyclonal, bs-5768R, Bioss antibodies, Woburn, MA), anti-CD56 antibody (monoclonal, CD56-504-L-CE, Leica-biosystems, Nussloch, Germany), and anti-SATB2 antibody (monoclonal, ab92446, Abcam, Cambridge, UK) according to the manufacturer’s instructions. Counterstain was performed with hematoxylin.

*Nucleic acid preparation*

We extracted genomic DNA and total RNA from the resected tumor specimen and peripheral blood mononuclear cells (PBMC) from the patient and total RNA from transfected HEK293T cells using a QIAamp DNA Blood Mini Kit and an RNeasy Mini Kit (QIAGEN, Hilden, Germany) according to the manufacturer’s instructions. We assessed the quality of the extracted RNA using an RNA ScreenTape and an Agilent 2200 TapeStation system (Agilent, Santa Clara, CA).

*RNA sequencing*

To prepare non-directional sequencing libraries according to the manufacturer’s instructions, we used an NEBNext Ultra RNA Prep Kit for Illumina with an NEBNext Poly(A) mRNA Magnetic Isolation Module or a NEBNext rRNA Depletion Kit (New England Biolabs). The prepared libraries were run on HiSeq 2500 with a 2 × 100-bp paired end-reads option. For analysis of the fusion gene, expression analysis and differential expression analysis, we used TopHat-Fusion, HTSeq, GFOLD and DESeq.

*Whole-exome sequencing*

Whole-exome sequencing (WES) of paired tumor-normal samples from the patient was perfomed. Exome capture from paired tumor reference DNA was performed by using a SureSelect XT target enrichment system and SureSelect Human All Exon v3 or v5 bait (Agilent, Santa Clara, CA), according to the manufacturer’s instructions. The prepared libraries were run on a HiSeq 2000/2500 sequencing system (Illumina, San Diego, CA). Candidate somatic mutations were detected using our in-house pipeline for WES (Genomon: http://genomon.hgc.jp/exome/) as previously described ^1^.

*Polymerase Chain Reaction*

Amplification of target regions containing break point of chromosomal structural variations from template genomic DNA was performed using SYBR Green Realtime PCR Master Mix (Toyobo, Osaka, Japan). Primer sequences are listed in supplemental table. Genomic DNA derived from non-PMT patient’s tissue was used as the negative control.

*Gene set enrichment analysis*

Gene set enrichment analysis (GSEA) was used for analyzing differential expression values calculated by GFOLD and the Molecular Signature Database (v6.1, http://www.broad.mit.edu/gsea/, accessed on 19/03/2018). We used the hallmark gene sets (50 gene sets), and considered a false discovery rate (FDR) of less than 0.1 as statistically significant.

*Cell Proliferation assay*

HEK 293T cells were transfected with mock or *NIPBL-BEND2* CSIV-CMV-IRES2-Venus vectors using ScreenFect A (Wako Chemicals, Osaka, Japan). 48 hours after transfection, 1 × 106 cells were plated to new 90 mm (57 cm2) dishes. 24 and 48 hours after plating, cell proliferation was measured by cell counting. MG63 cells were transfected with lentiviral particles produced using mock or *NIPBL-BEND2* CSIV-CMV-IRES2-Venus vector, Lentiviral High Titer Packaging Mix (TaKaRa Bio, Ohtsu, Japan), and Lenti-X cell line (TaKaRa Bio) at a multiplicity of infection of 3. 48 hours after transfection, cells were sorted based on green fluorescence using a FACSAria2 cell sorter. After cell sorting, *NIPBL-BEND2* transfected MG63 cells and mock MG63 cells were seeded in 96‐well plates at 2 × 10^3^/well in medium containing 10% FBS and allowed to adhere for 6 hours. After 24 and 48 hours, cell proliferation was measured using the (3‐(4,5‐dimethylthiazol‐2‐yl)‐5‐(3‐carboxymethoxyphenyl)‐2‐(4‐sulfophenyl)‐2H‐tetrazolium, inner salt; MTS) colorimetric assay (Promega).

Ethics statement

This study was approved by the Institutional Review Board at the investigator's institution (approval number: 2014-0181). Informed consent was obtained from the patient and parent.

Reference

[1] Yoshida K, Sanada M, Shiraishi Y, Nowak D, Nagata Y, Yamamoto R, Sato Y, Sato-Otsubo A, Kon A, Nagasaki M, Chalkidis G, Suzuki Y, Shiosaka M, Kawahata R, Yamaguchi T, Otsu M, Obara N, Sakata-Yanagimoto M, Ishiyama K, Mori H, Nolte F, Hofmann WK, Miyawaki S, Sugano S, Haferlach C, Koeffler HP, Shih LY, Haferlach T, Chiba S, Nakauchi H, Miyano S, Ogawa S: Frequent pathway mutations of splicing machinery in myelodysplasia. Nature 2011, 478:64-9.

Supplemental Figure 1. A macroscopic image of the tumor to be resected as en bloc during surgery.


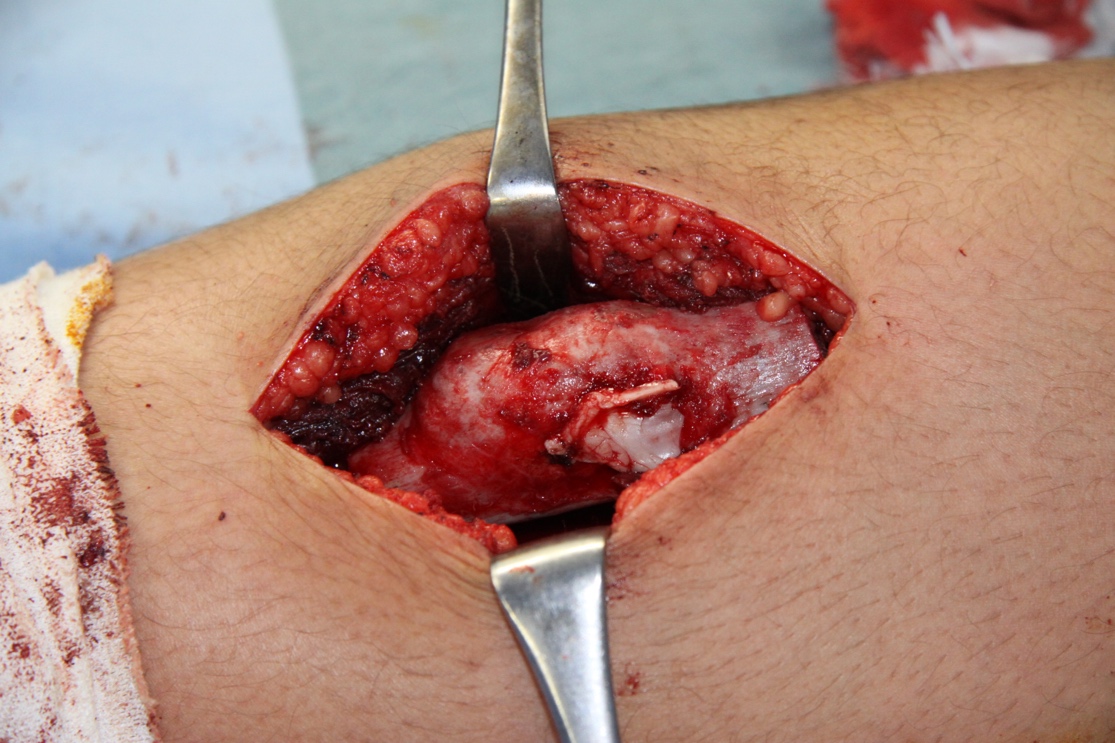


Intraoperative macroscopic image of en-bloc resection. The diaphysis of the fibula was cut at the proximal (right side) and the distal (left side) of the tumor. Bone cement was inserted in the fenestration for intraoperative diagnosis. Supplemental Figure 2. H & E stained specimen in x200 magnification and

CD56, SATB2 immunohistostained specimen in x200, 400 magnification

**
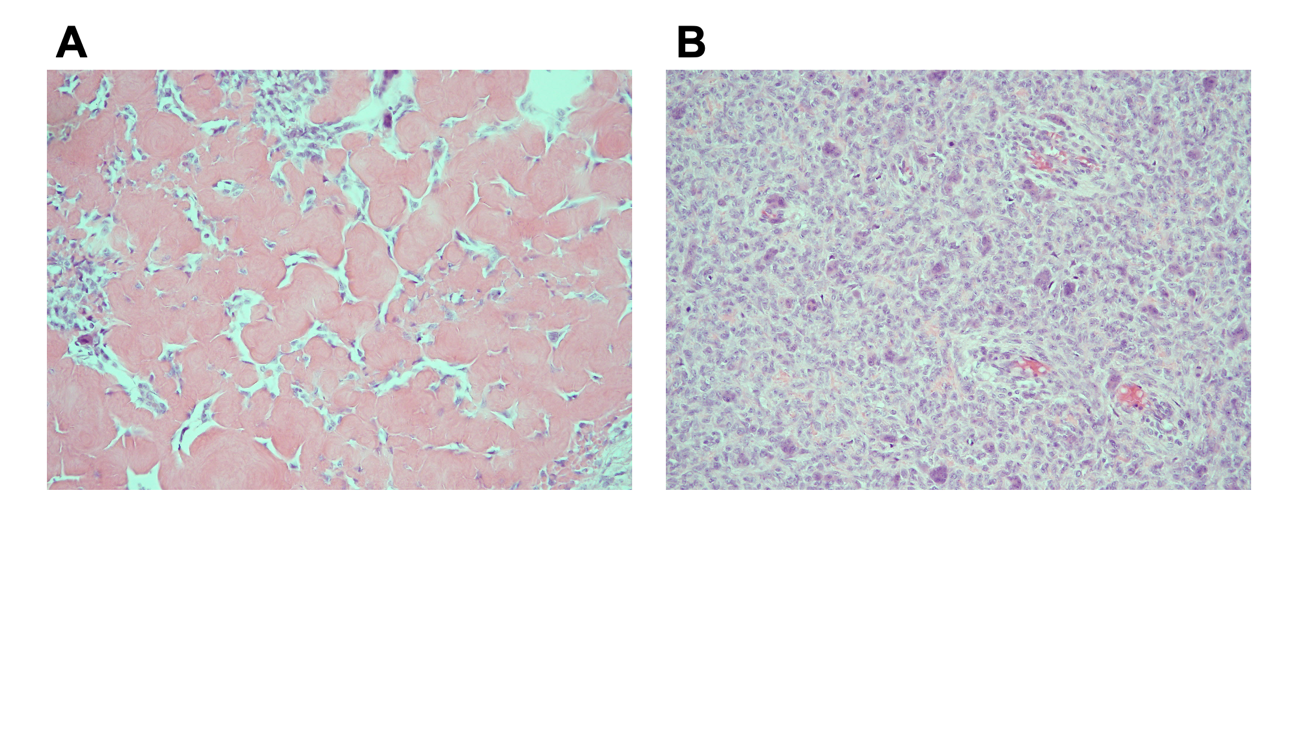
**

**
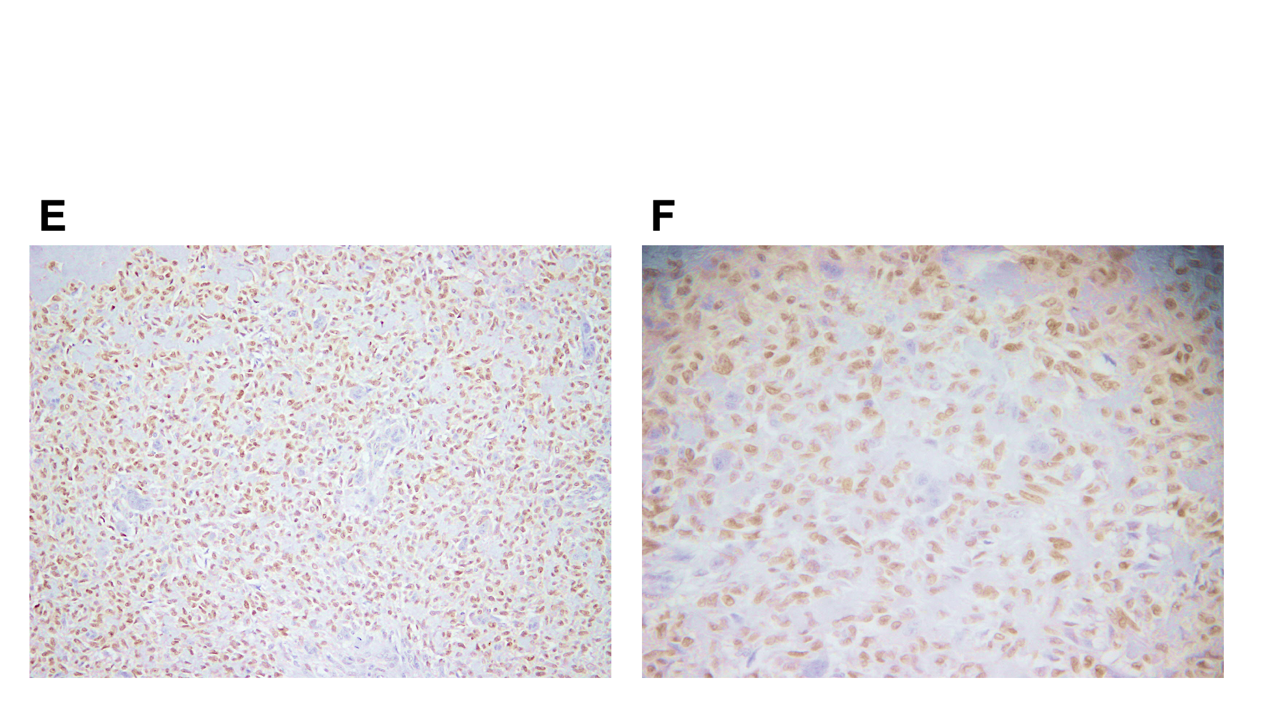
**

Two typical pathological findings in tumors at 200x magnification; (A) Osteoblast-like tumor cells are scattered in the background of the eosinophilic osteoid matrix. There are no malignancy findings such as atypical cells. (B) Reactive osteoclastic giant cells exist in the background of histiocyte-like mononuclear cells with high cellularity. There are no malignancy findings and few mitoses.

(C, D) The tumor cells are diffusely positive on the cell membrane for CD56 expression.

(E, F) The tumor cells show strong diffuse immunostaining with SATB2.

Original magnification is x200, x400 respectively.

Supplemental Figure 3. Detection of *NIPBL-BEND2* fusion


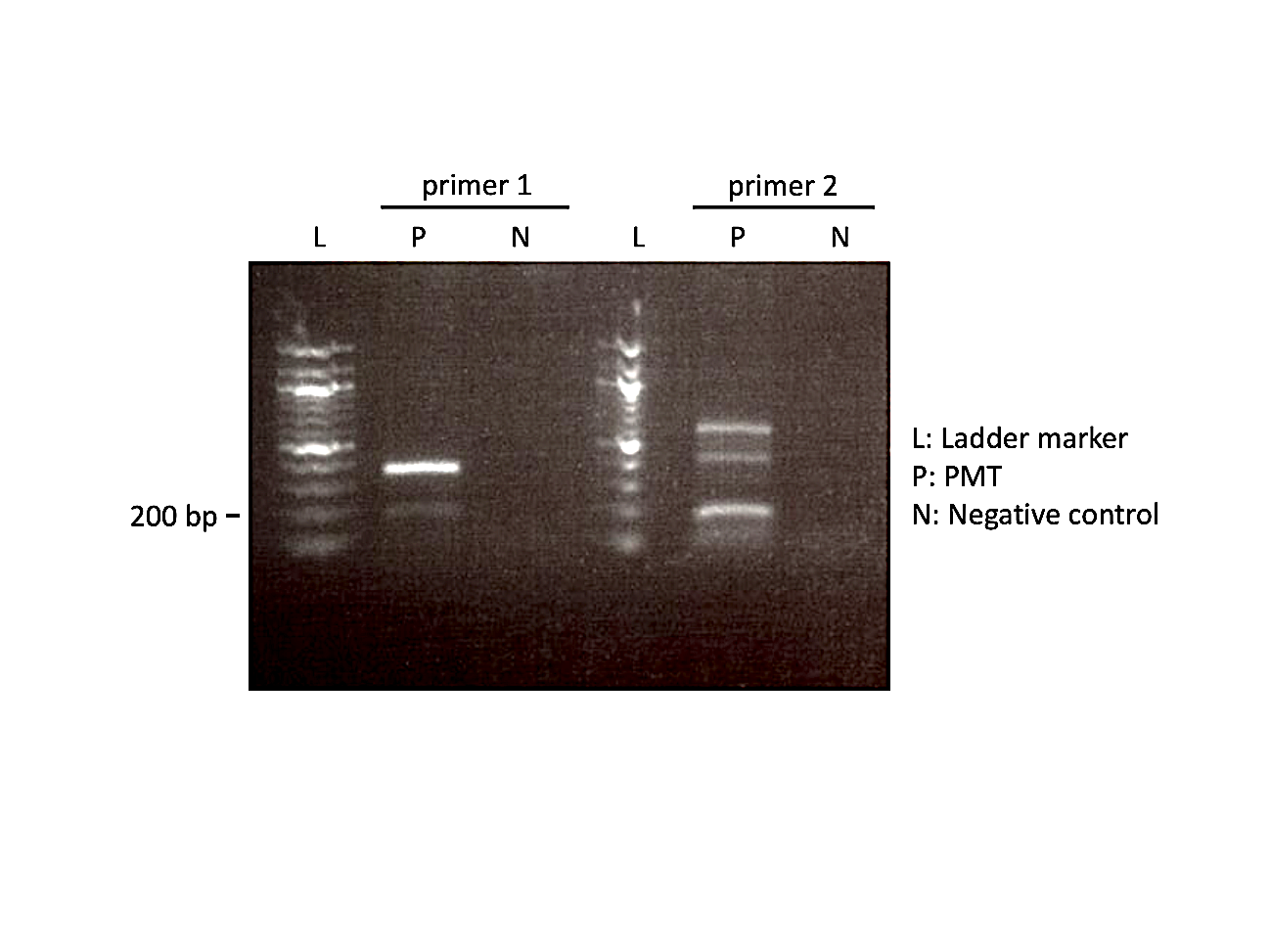


Detection of *NIPBL-BEND2* fusion based on genomic DNA. The expected product lengths for primer sets 1 and 2 are 191 and 199, respectively.

Supplemental Table 1. Primers used to validate the *NIPBL-BEND2* fusion gene

| Name | F/R | Sequences (5' - 3') |
| --- | --- | --- |
| primer 1 | Forward | GGGCACTCGTATTTCCCGAT |
|  | Reverse | AGTCCATACGCCCCACAAAG |
| primer 2 | Forward | GGGCACTCGTATTTCCCGATA |
|  | Reverse | CAGTGCCTAGTCCATACGCC |

Supplemental Table 2. FPKM score of FGF23, FGFR1, MEPE, SFRP4, and KL in mRNA from the tumor tissue

| gene_id | locus | FPKM |
| --- | --- | --- |
| FGF23 | chr12:4477392-4488894 | 410.166 |
| SFRP4 | chr7:37945534-37956525 | 935.196 |
| MEPE | chr4:88742549-88767968 | 2587.24 |
| FGFR1 | chr8:38268655-38326352 | 219.244 |
| KL | chr13:33590570-33640282 | 51.0994 |
